# *N*-Palmitoylethanolamine Maintains Local Lipid Homeostasis to Relieve Sleep Deprivation–Induced Dry Eye Syndrome

**DOI:** 10.3389/fphar.2019.01622

**Published:** 2020-01-28

**Authors:** Qi Chen, Chunyan Ji, Ruihe Zheng, Longhe Yang, Jie Ren, Yitian Li, Yun Han, Pan Zhou, Zuguo Liu, Yan Qiu

**Affiliations:** ^1^ Eye Institute of Xiamen University, Fujian Provincial Key Laboratory of Ophthalmology and Visual Science, School of Medicine, Xiamen University, Xiamen, China; ^2^ Department of Ophthalmology, Xiang’an Hospital of Xiamen University, Xiamen, China; ^3^ Xiamen University Affiliated Xiamen Eye Center, Xiamen, China; ^4^ Engineering Research Center of Marine Biological Resource Comprehensive Utilization, Third Institute of Oceanography, State Oceanic Administration, Xiamen, China

**Keywords:** palmitoylethanolamide, lacrimal gland, lipid accumulation, dry eye, peroxisome proliferator-activated receptor-α

## Abstract

Sleep loss is a key factor associated with dry eye. Use of a “stick over water” mouse model revealed that sleep deprivation induces accumulation of lipids, hypertrophy, and dysfunction of the lacrimal gland. These changes result in decreased tear production and dry eye clinical signs. The specific pathophysiological mechanisms that contribute to dry eye remain unclear. In this study, we found that sleep deprivation decreased endogenous lipid palmitoylethanolamide (PEA) expression in the lacrimal gland. The reduced expression was mainly attributed to the decreased expression of *N*-acylated phosphatidylethanolamine–phospholipase D, the synthetic enzyme of PEA. Exogenous PEA treatment restored local lipid metabolism homeostasis in the lacrimal gland. This change was accompanied by reduced lipid deposition, maintenance of the endoplasmic reticulum and mitochondrial morphology, and improved acinar cell secretory function. PEA treatment also prevented damage to corneal barrier function and improved the dry eye clinical signs caused by sleep deprivation. The nuclear receptor peroxisome proliferator-activated receptor-α (PPAR-α) was found to mediate the PEA-associated improvements. We describe here for the first time that PEA is involved in sleep deprivation–induced lacrimal gland pathogenesis and dry eye development. PEA and its metabolizing enzymes may serve as adjunctive therapeutic targets for treatment of dry eye.

## Introduction

Eye disease accompanied by discomfort or chronic pain (e.g., dry eye and chronic conjunctivitis) can affect sleep quality *via* the trigeminal nerve pathway. However, sleep disorders also cause eye problems, especially dry eye disease (DED). Sleep, age, gender, and medication are key risk factors associated with DED (Ahn et al., 2014). However, only a limited number of population-based studies and animal-based experiments have examined the associations between sleep disorders and dry eye.

Dry eye is a multifactorial disease of the ocular surface characterized by a loss of homeostasis of the tear film (Craig et al., 2017). Accompanying ocular signs include tear film instability, hyperosmolarity, inflammation, ocular surface damage, neurosensory abnormality, and dysfunction of the main lacrimal gland (LG). In 2015, Korean researchers published data from 15,878 adult subjects to assess the correlation between sleep duration and dry eye (Lee et al., 2015). The results indicated that a higher dry eye prevalence was present in groups with severe short sleep duration (< 4 h). Our preliminary studies using mouse models found that sleep deprivation (SD) causes ocular surface disorders, including aqueous tear deficiency, corneal epithelial defects, corneal sensitivity, and apoptosis (Li et al., 2018). Hypertrophy and increased lipid accumulation are compensatory LG responses. Abnormal superficial corneal epithelial cell microvilli morphology is another main cause of SD-induced dry eye (SDE)–like symptoms. This morphological change is associated with peroxisome proliferator-activated receptor-α (PPAR-α)–mediated lipid metabolism abnormalities (Tang et al., 2018).

Fatty acid ethanolamides (FAEs) are members of a class of endogenous bioactive lipid mediators with a core chemical *N*-acylethanolamine structure (Bottemanne et al., 2018). Principal FAE family members include the endocannabinoid anandamide (AEA), and its congeners oleoylethanolamide (OEA) and palmitoylethanolamide (PEA). The anti-cancer and anti-depression effects of AEA are mediated by activation of cannabinoid receptors and vanilloid receptors (Deutsch and Chin, 1993). OEA and PEA act as endogenous agonists of PPAR-α and exert anorexic, anti-inflammatory, and analgesic effects (Yang et al., 2015). Endogenous PEA is generated from *N*-acylated phosphatidylethanolamine (NAPE; a major membrane phospholipid) by the enzyme NAPE–phospholipase D (NAPE-PLD). PEA is selectively hydrolyzed by lysosomal *N*-acylethanolamine–hydrolyzing acid amidase (NAAA) (Ueda et al., 2010).

Study results suggest that FAEs regulate the sleep–wake cycle (Prospéro-García et al., 2016). Microinjection of PEA or OEA into the lateral hypothalamus or dorsal raphe nucleus of rats causes increases in wakefulness and decreases in slow-wave sleep periods (Murillo-Rodríguez et al., 2011). However, to our knowledge, there are few published reports on FAE release in patients with sleep disorders. In a 2008 study, FAE levels were measured in the cerebrospinal fluid of 20 healthy volunteers, before and after a 24 h SD period (Koethe et al., 2009). The results indicated that SD increased OEA levels in human cerebrospinal fluid but not in serum. This difference might indicate the presence of a neuroprotective signal during SD. Endogenous AEA levels remained unaffected, and PEA levels were not detected. In another study, differences in plasma FAE levels between the summertime period and the torpor period of hibernation were examined in *Marmota monax* (groundhog) (Vaughn et al., 2010). During hibernation, OEA levels were significantly decreased, and the concentration of PEA was significantly increased. The elevated PEA was assumed to contribute to suppression of immune function during long-term sleep. The specific roles and functions of these FAEs in sleep disorders have not been determined.

PEA has been found in different human tissues, including ocular system tissue. Recently, PEA has been prepared as a novel nanostructured lipid carries (NLCs) formulation for ocular surface administration, which was considered beneficial for management of retinal disease (Puglia et al., 2018). In this study, we found that the levels of PEA expression and the homeostasis of endogenous lipids in the lacrimal system were altered after SD. These changes likely were associated with DED progression. The aim of this study was to investigate the effects of PEA on re-establishment of endogenous lipid homeostasis and management of dry eye syndrome using an SD-induced mouse model.

## Materials and Methods

### Materials

The PEA and PPAR-α antagonist MK886 were obtained from Sigma-Aldrich [Shanghai, China; purity ≥98%, measured using high-performance liquid chromatography (HPLC)]. All other reagents were also purchased from Sigma-Aldrich (Shanghai, China) and were the highest grade commercially available, unless otherwise indicated.

### Animal Experiments

#### SD Mouse Model

Adult male C57BL/6J mice (20–22 g) were purchased from Shanghai Laboratory Animal Center (SLAC, Shanghai, China). PPAR-α knockout (PPAR-α^−^^/^^−^) mice with a C57BL/6J background were purchased from the Jackson Laboratory (Bar Harbor, ME, USA) and were bred at the Laboratory Animal Center of Xiamen University. Before using in the experiment, all mice adapted to the environment for a week, and were examined to ensure ocular surface health. SD was performed using a “stick over water” method in a standard laboratory setting, as previously described (Li et al., 2018). Two mice were housed per cage in standard laboratory conditions. In the middle of the cages, two 6-mm-diameter sticks were added, 4 cm above the bottom, and 6 cm apart. Water was added into the cage to 1 cm below the sticks. Animals were fed a standard diet and tap water ad libitum. When the mice were sleepy, they would lose balance and fall into the water, which awakened the animals. The SD time lasted for 20 h. Animals were observed once every 4 h to prevent death from drowning. After the SD period, each animal was gently dried using a hair dryer and blotting paper, and was transferred to its dry home cage. Sleep time was arranged in the noon, and any disturbance was forbidden. The room temperature was kept at 25 ± 1°C with a 12 h light:12 h dark cycle. Each animal was anesthetized using sodium pentobarbital [50 mg/kg, intraperitoneal (i.p.) route] before being humanely killed.

All animal experimental protocols were in accordance with the Association for Research in Vision and Ophthalmology Statement for the Use of Animals in Ophthalmic and Vision Research guidelines and were approved by the Experimental Animal Ethics Committee of Xiamen University.

Each experimental animal was randomly assigned to one of seven treatment group conditions: 1. *SD group* (*n* = 57). Animals were sleep-deprived 20 h per day for 5 or 10 days and given water ad libitum. After SD, mice were transferred to their dry home cages, and disturbance was forbidden. 2. *Control (Ctrl) group* (*n* = 46). Animals were kept in standard home cages without SD or any other treatment. 3. *Vehicle (Veh) group* (*n* = 46). Animals were sleep-deprived for 20 h per day for 10 days. SD-induced mice were injected (i.p. route) with vehicle solvent (saline with 10% polyethylene glycol-400 and 10% Tween-80) twice a day (8 a.m. and 8 p.m.), from day 6 until day 10. 4. *PEA treatment (PEA) group* (*n* = 56). Animals were sleep-deprived for 20 h per day for 10 days. SD-induced mice were treated with PEA (3, 10, 30 mg/kg; i.p. route) twice a day (8 a.m. and 8 p.m.), from day 6 until day 10. 5. *MK886 treatment (MK886) group* (*n* = 10). SD-induced mice were treated with PPAR-α antagonist MK886 (15 mg/kg; i.p. route) twice a day (8 a.m. and 8 p.m.), from day 6 until day 10. 6. *PEA treatment with MK886 (PEA + MK886) group* (*n* = 10). SD-induced mice were injected with PPAR-α antagonist MK886 (15 mg/kg; i.p. route) twice a day (8 a.m. and 8 p.m.), from day 6 until day 10. Twenty minutes later, the mice were treated with PEA (30 mg/kg, i.p.). 7. *PPAR-α knockout mice (PPAR-α^−^^/^^−^) group* (*n* = 11). Animals were kept in standard home cages without SD, and the other treatment was consistent with the description above.

#### Lipid Extraction and Analysis

A total of 20–30 mg frozen LG tissue samples were homogenized in 2 ml mixtures of methanol/water (1:1, vol:vol) containing 1 nM heptadecenoylethanolamide (17:1 FAE) as an internal standard. Endogenous FAEs were extracted with 4 ml chloroform, vortexed for 1 min, and then centrifuged for 10 min at 4°C and 3000 × *g*. Most of the FAEs were separated during the chloroform phase. The organic phase was collected and evaporated to dryness under an N_2_ stream using a nitrogen evaporator (Beijing, TongtaiLian Technology Co., Ltd.). The dried lipids were resuspended in 1 ml chloroform. Solid-phase extraction was performed by loading the lipid solution into its respective silica gel column. The samples were eluted with 3 ml chloroform/methanol (9:1, vol:vol). The purified FAEs were dried under an N_2_ stream, and then redissolved in 100 μl methanol for HPLC/mass spectrometry (MS^2^) analyses.

The samples were analyzed using an 1100-HPLC system (Agilent, Shanghai, China) equipped with an Applied Biosystems 3200 triple-quadrupole linear ion trap mass spectrometer (Applied Biosystems, Concord, Canada). The elution gradient for the mobile phase was: 90% methanol for the first 5 min, followed by a linear gradient of 90% to 100% methanol for 5 min, and then maintenance at 100% methanol for 20 min. The HPLC flow rate was 0.7 ml/min. The column temperature was 40°C. Multiple reaction monitoring transitions in positive ion atmospheric pressure chemical ionization mode were performed for ion detection: AEA, *m/z* 348.00/62.00; OEA, *m/z* 326.10/62.00; PEA, *m/z* 300.2/62.00; and 17:1 FAE, *m/z* 313.1/62.00.

#### Measurement of Tear Production

Phenol red cotton thread (Zone-Quick; Yokota, Tokyo, Japan) was used to absorb aqueous tear production for measurement. Each test was conducted in the standard environment at the same time by the same operator. Briefly, the mouse lower eyelid was pulled down gently to expose the conjunctival sac. The thread was then placed on the lower conjunctival fornix near the lateral canthus at about one-third of the length of the lower lid. After 15 s, the thread was removed, and the red wetted length was measured in millimeters. After the test, both eyes were closed to avoid excessive exposure leading to ocular surface irritation.

#### Periodic Acid–Schiff Staining

Eyes with ocular adnexa were surgically excised and fixed in 4% paraformaldehyde for 24 h. After dehydration in gradient alcohol, the tissues were embedded in paraffin and cut into 8 µm sections. Dimethylbenzene was used for deparaffinization. After soaking in double-distilled water for 10 min, the tissue sections were stained with periodic acid for 10 min. Schiff reagent (Leica Biosystems, Nussloch, Baden, Germany) was used to stain the sections for 8 min after rinsing with double-distilled water for 10 min. Subsequently, the sections were soaked in double-distilled water for another 3 min and were stained with hematoxylin for 35 s. During counting, the goblet cells in the superior and inferior conjunctivae were viewed using a light microscope (Eclipse 50i, Nikon, Tokyo, Japan).

#### Hematoxylin–Eosin Staining

The LG samples were placed in 4% paraformaldehyde at 4°C for fixation. After dehydration and clearing, the tissues were embedded into paraffin wax and cut into 6 µm sections. The tissue sections were deparaffinized, rehydrated, and stained for morphological examination using hematoxylin and eosin. In order to ensure the reliability of the data, we kept the placement position consistently when the LG is embedded. The slice is guaranteed to have the same cross-sectional direction when sectioning. In addition, three slides were taken from the front, middle, and last parts of each sample during sectioning. The morphological differences of LGs were compared by the ratio of the mean area of individual acini in each group to the control group of acinar area under the same magnification micrographs.

#### Oil Red O Staining

LG tissues were collected from the mice and immediately flash-frozen in optical coherence tomography (OCT, SAKURA Tissue-Tek, Torrance, CA, USA) with liquid N_2_. The tissues were cut into 6-µm-thick sections using a cryostat microtome (CM1850 UV, Leica Microsystems, Wetzlar, Germany). The sections were air-dried for 10 min at room temperature, fixed with 10% buffered formalin for 20 min, and then washed with 1× PBS solution three times (5 min each time). Approximately 1 ml oil red O (ORO) working solution was added to completely cover the sections, and they were incubated at room temperature for approximately 10 min. The sections were submerged in hematoxylin for 45 s, and thereafter rinsed under running water for 10 min. The ORO stain results were examined using light microscopy (Eclipse 50i, Nikon, Tokyo, Japan).

#### Biological Tissues Preparation for Ultrastructural Study

The LG tissues were immediately collected after the mice were euthanized; the tissues were fixed in a 2.5% solution of glutaraldehyde buffer to preserve the fine structure. The tissues were cut into small pieces (1 × 1 × 2 mm) and fixed overnight at 4°C. They were then washed with three times (15 min each time) with cold PBS and secondarily fixed in 1% osmic acid for 2 h at 4°C. After dehydration with gradient ethanol and infiltration with Spurr’s resin, the fixed tissues were encased in hardened blocks to be thin-sectioned using an ultramicrotome (Leica EM UC6 and Reichert Ultracut S, Leica Microsystems GmbH, Wetzlar, Germany). The thin LG sections were counterstained and then examined and photographed using a transmission electron microscope (TEM, JEM2100HC, JEOL, Tokyo, Japan).

#### Measurement of Corneal Permeability

Corneal epithelial permeability was assessed using Oregon Green Dextran (OGD, 70 kDa, Invitrogen, Eugene, Oregon, USA) for both eyes in the mice from each group. Briefly, 0.5 μl OGD (50 mg/ml) was applied to the mouse cornea for 1 min. The mice were then euthanized, and the eyes were rinsed with 1 ml saline (five times). Digital pictures were photographed using a multizoom fluorescence microscope (470 nm excitation and 488 nm emission wavelengths, AZ100, Nikon, Tokyo, Japan). The mean fluorescence intensity of corneal OGD staining in the digital images was calculated for a 3-mm-diameter circle in a fixed central cornea region using analysis software (NIS Elements, version 4.1, Nikon, Melville, NY, USA).

#### Measurement of Corneal Sensitivity

Corneal sensitivity was quantified by using a Cochet-Bonnet esthesiometer (Luneau, Paris, France). The pressure applied to the center of the cornea was proportionate to the inverse of the esthesiometer nylon filament length. That is, the longer the nylon filament, the lower the pressure exerted on the apex of the cornea. When the force exerted perpendicularly to the center of the cornea was perceived by the mouse, a blink response was evoked. Determination of the threshold to stimulation was made using a 0.12-mm-diameter filament with a maximum length of 60 mm. Five consecutive stimulus presentations were conducted for each eye. When three or more blink responses were observed in the test, a positive result was considered, and the length was recorded. If the threshold could not be detected, the nylon filament length was reduced in 5 mm increments to increase stimulus intensity.

#### Real-Time Quantitative PCR

The total RNA was extracted from LG tissues using TriPure isolation reagent (Roche, Shanghai, China) according to the manufacturer’s protocol. Total RNA was inverted into cDNA using a ReverTra Ace qPCR RT kit (TOYOBO, Shanghai, China). The amount of cDNA required for running PCR was added according to the Fast Start Essential DNA Green Master (Roche, Shanghai, China) instructions. Quantification of mRNA expression was performed using SYBR Premix Ex TaqTMII (Takara, Dalian, China) and a LightCycler 96 System (Roche, Shanghai, China). The housekeeping gene glyceraldehyde 3-phosphate dehydrogenase (*GAPDH*) was used as an internal standard to normalize the relative expression of target genes.

The primers were designed and synthesized based on gene sequences available in the GenBank database. The mouse gene sequences were: NAPE-PLD, forward primer (F): 5’-TGGCTGGGACACGCG-3’, reverse primer (R): 5’-GGGATCCGTGAGGAGGATG-3’; fatty acid amide hydrolase (*FAAH*), (F): 5’-GCCTCAAGGAATGCTTCAGC-3’, (R): 5’-TGCCCTCATTCAGGCTCAAG-3’; *NAAA*, (F): 5’-GACTCCGCCTCTCTTCAACG-3’, (R): 5’-ACCATCCCGAGTACCCACTG-3’; *PPAR-α*, (F): 5’-AGAGCCCCATCTGTCCTCTC-3’, (R): 5’-ACTGGTAGTCTGCAAAACCAAA-3’; glyceraldehyde-3-phosphate dehydrogenase (*GAPDH)*, (F): 5’-ACCACGAGAAATATGACAACTCCC-3’, (R): 5’-CCAAAGTTGTCATGGATGACC-3’; stearoyl-CoA desaturase-1 (*SCD1)*, (F): 5’-ATCGCCCCTACGACAAGAAC -3’, (R): 5’-AACTCAGAAGCCCAAAGCTCA-3’; carnitine palmitoyl-transferase 1α (*CPT1α*), (F): 5’-GACTCCGCTCGCTCATTCC-3’, (R): 5’-CACCAGTGATGATGCCATTCTTG-3’; lipoprotein lipase (*LPL*), (F): 5’-AGGGCTCTGCCTGAGTTGTA-3’, (R): 5’-AGAAATCTCGAAGGCCTGGT-3’; acyl-CoA oxidase 1 (*ACOX1*), (F): 5’-TCGAAGCCAGCGTTACGAG -3’, (R): 5’-GGTCTGCGATGCCAAATTCC-3’; acetyl coenzyme A carboxylase 1 (*ACC1)*, (F): 5’-TGCAGGTATCCCCACTCTTC-3’, (R): 5’-TTCTGATTCCCTTCCCTCCT-3’; acetyl coenzyme A carboxylase 2 (*ACC2*), (F): 5’-TTTCTGATGTGCTGGAATGG-3’, (R): 5’-GACTGTGTGTGCTCGTGGTT-3’.

### Statistical Analysis

All samples were scored or observed blindly and independently by at least two investigators. Each experiment was sequentially performed at least three times. The data were analyzed using GraphPad Prism version 5.0 (GraphPad Software, San Diego, CA, USA). The results were expressed as mean ± standard error of the mean values. Statistical comparisons were performed appropriately using unpaired *t*-tests or one-way analysis of variance followed by *post-hoc* tests. A result was considered statistically significant if the associated *P*-value was <0.05.

## Results

### SD Reduced LG Tissue PEA Levels

**Figure 1:**
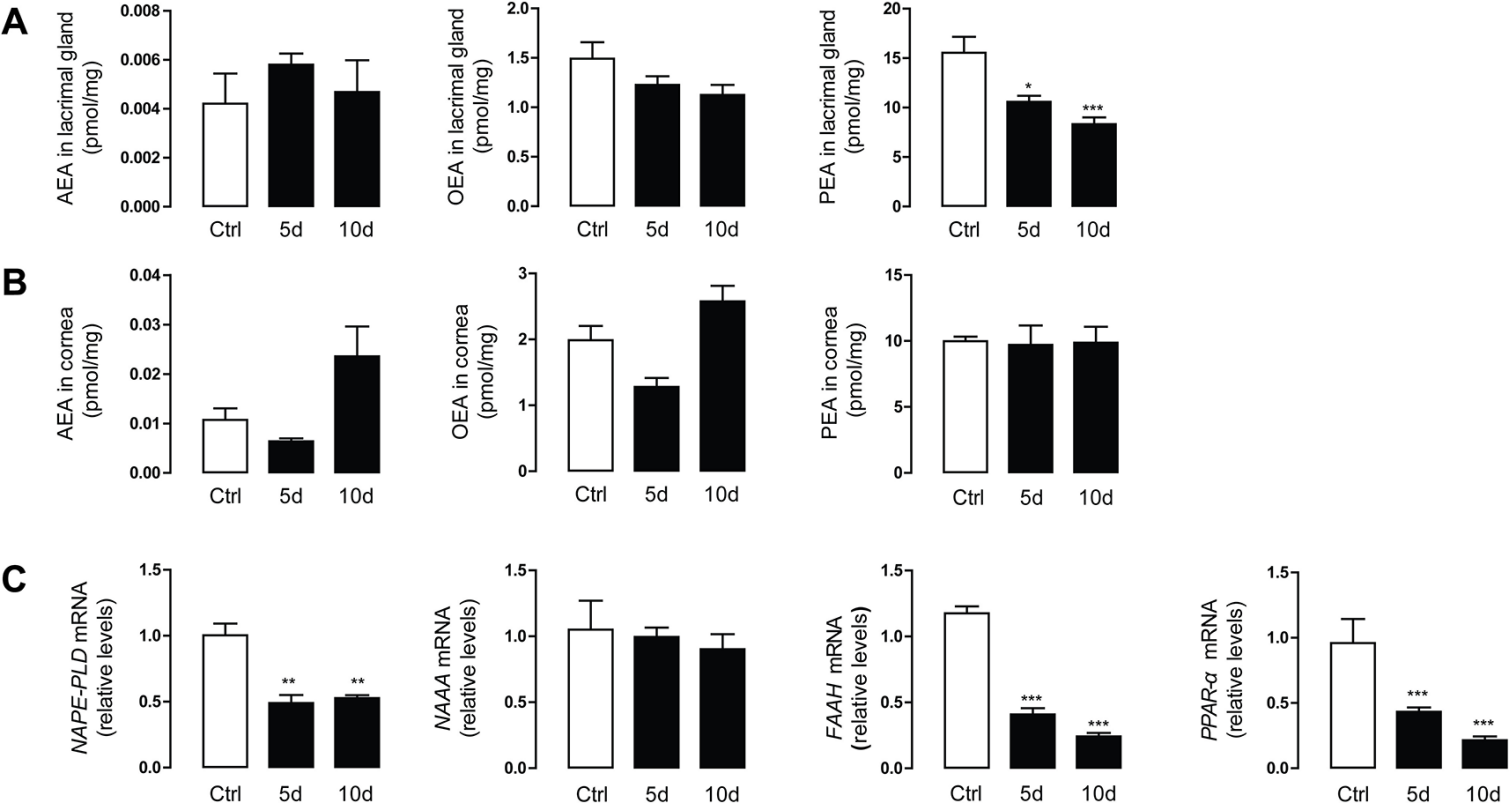
Sleep deprivation (SD) reduced the lacrimal gland (LG) levels of palmitoylethanolamide (PEA). Levels of anandamide (AEA), oleoylethanolamide (OEA), and PEA in LG tissue **(A)** and corneal tissue **(B)**. Control group (Ctrl, open bars), SD-induced mice model for 5 and 10 days (5d, and 10d, respectively, solid bars). Bottom panel showed the mRNA expression levels of FAE metabolic enzymes and peroxisome proliferator-activated receptor-α (PPAR-α) in LG tissue **(C)**. *, P < 0.05; **, P < 0.01; ***, P < 0.001 vs. control; unpaired *t*-test, n = 5.

To examine the role of endogenous lipids during the progression of SDE, the levels of FAEs were first determined and analyzed in the LG and corneal tissues of the mice using HPLC/MS^2^. The mean endogenous PEA levels in LG tissue significantly decreased after 5 and 10 days of SD (10.58 ± 1.39 and 8.32 ± 1.68 pmol/mg, respectively) in a time-dependent manner, compared with control group mice (15.55 ± 2.77 pmol/mg, **Figure 1A**). However, SD did not affect the endogenous OEA and AEA levels in LG tissue. SD did not markedly alter the levels of PEA, OEA, or AEA in corneal tissue (**Figure 1B**).

To understand the molecular mechanism underlying the alteration in FAEs, we used quantitative PCR to examine mRNA expression of the enzymes involved in FAE biosynthesis and degradation (e.g., NAPE-PLD, NAAA, and FAAH). Compared with the control group, NAPE-PLD and FAAH mRNA expression in the SD group decreased significantly, accompanied by downregulation of the gene expression of *PPAR-α*. There was no difference in the expression level of NAAA, the PEA degrading enzyme, between the treatment group and the control group (**Figure 1C**).

### Dose-Dependent Exogenous PEA Amelioration of Lacrimal System Damage in Wild-Type Mice

**Figure 2:**
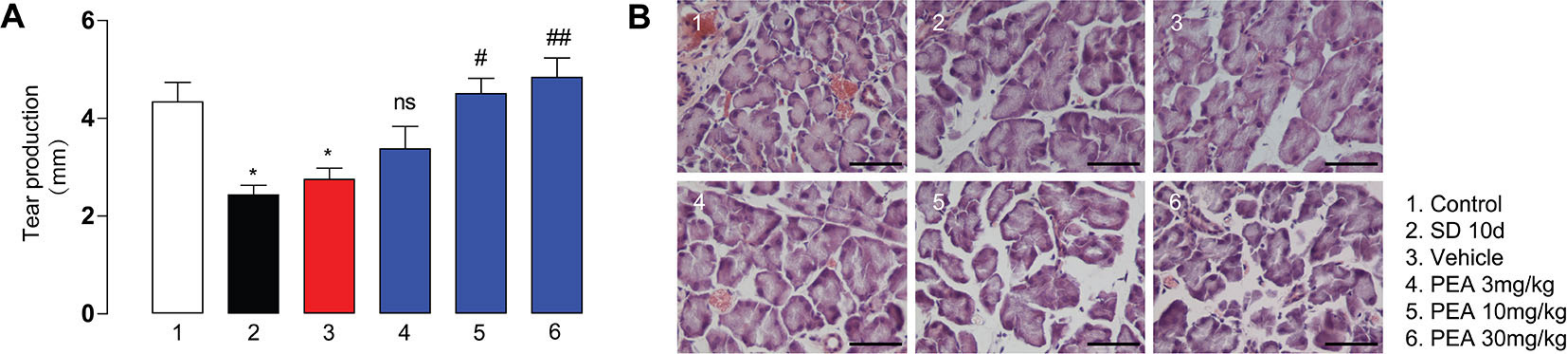
PEA treatment in SD-induced mice dose-dependently increased tear secretion **(A)** and reduced LG acini size **(B)**. *, P < 0.05 vs. control group (1); ^##^, P < 0.01; ^###^, P < 0.001 vs. vehicle group (3), n = 5.

Phenol red thread measurement of tear secretion revealed that compared with the control group (4.3 ± 0.9 mm), the tear secretion in the 10-day SD group (2.4 ± 0.5 mm) was notably reduced. Tear secretion in the vehicle group with solvent administration remained decreased. There was no remarkable difference between the low-dose PEA (3 mg/kg) treatment group and the vehicle group (3.4 ± 1.3 vs. 2.7 ± 0.6 mm). Middle- and high-dose PEA treatment significantly increased tear secretion to normal levels (4.5 ± 1.1 mm for 10 mg/kg PEA and 4.8 ± 1.4 mm for 30 mg/kg PEA, **Figure 2A**). This result suggested there was an overall improvement in lacrimal system function.

The LG consists of acinar cells that are generally round or irregular shapes and contain vesicles. The hematoxylin and eosin staining results indicated that SD induced a consistent increase in acinar volume; the acinar cell vesicles increased in size and expanded in volume (**Figure 2B**). PEA administration (10 and 30 mg/kg) reduced acinar cell size to the lean phenotype and prevented LG hypertrophy in the SD-induced mice (**Figure 2B**). The number of vesicles in the acinus also decreased in the PEA treatment groups. However, low-dose PEA application (3 mg/kg) did not significantly decrease the volume of acinar cells and the number of vesicles induced by SD. The high dose of PEA (30 mg/kg) was selected for subsequent pharmacodynamics and mechanism studies.

### PEA Treatment Reduced Lipid Accumulation in LG Tissue

**Figure 3:**
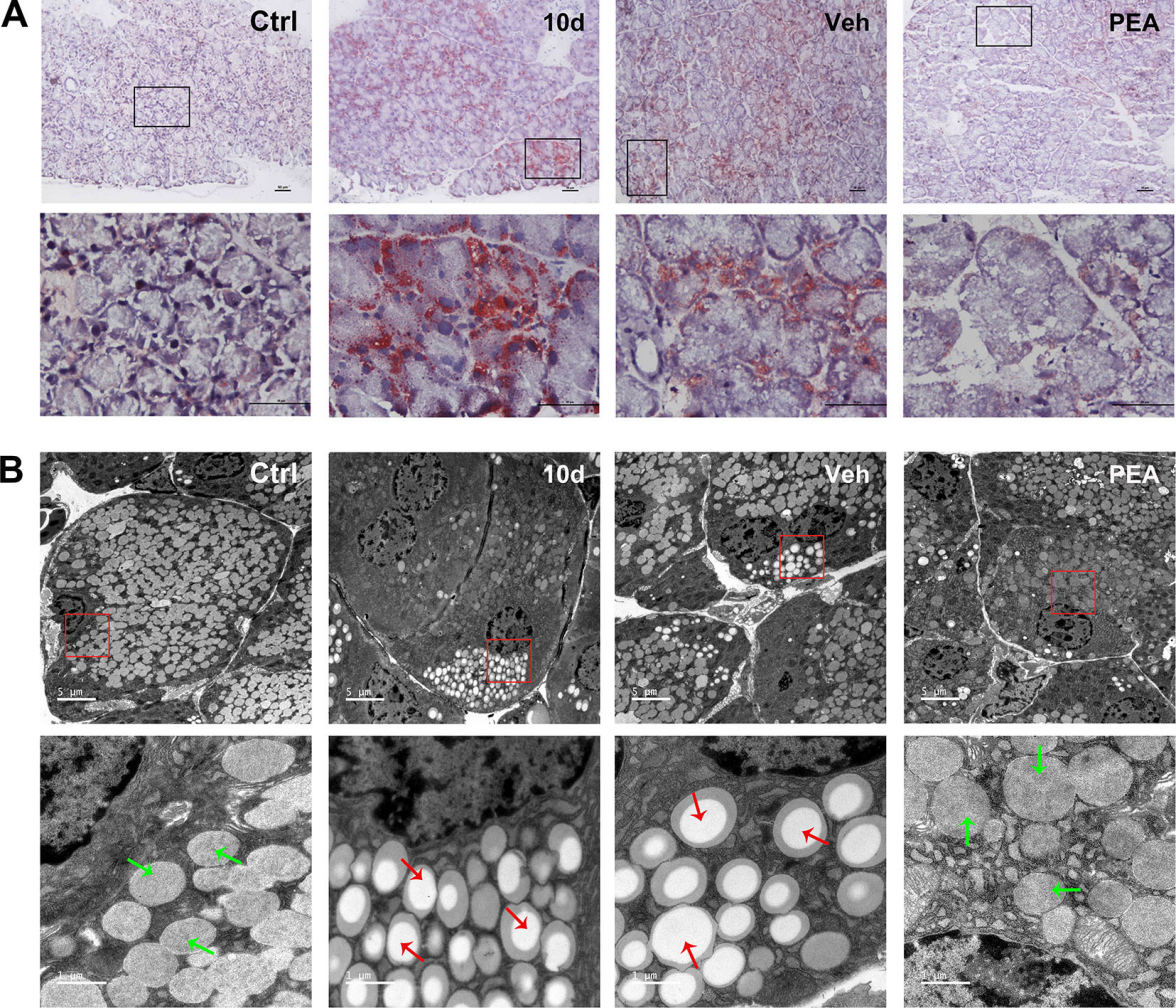
PEA treatment reduced lipid accumulation in SD-induced mice LGs. Oil red O staining results indicated that PEA reduced lipid deposition in SD-induced mice LG **(A)**, scale bars: 50 μm. Ultrastructural analysis under transmission electron microscopy revealed that PEA decreased lipid droplets accumulation close to the LG acinar cell nucleus **(B)**. Lipid droplets (red arrows), secretory granules (green arrows).

ORO staining was performed to assess the expression of neutral lipids, including triglyceride and cholesteryl oleate in the LG. The ORO results indicated that detectable lipid droplets began to accumulate in the LGs as early as the second day after acute SD (data not shown). The severity was further aggravated with the increased continuation of sleep loss. The lipid deposition was mainly located close to the cell nucleus in the cytoplasm and was distributed in the matrix between acinar cells. After 5 days of PEA treatment, the lipid droplet accumulation in LG tissue was significantly reduced, compared with the SD and vehicle groups (**Figure 3A**).

Ultrastructural analysis using TEM also revealed the presence of lipid droplet accumulation, which was located close to cell nuclei and basal membranes in the SD group mice (**Figure 3B**). Red arrows indicated mostly heterogeneous lipid-rich core regions of lipid droplets. Similar results occurred in the solvent vehicle group. In the control and PEA groups, only a few lipid droplets were close to the nucleus; secretory granules (green arrows) were also usually present in these areas. These results indicated that PEA administration resulted in a notable reduction in lipid accumulation, which was consistent with the ORO staining results.

**Figure 4:**
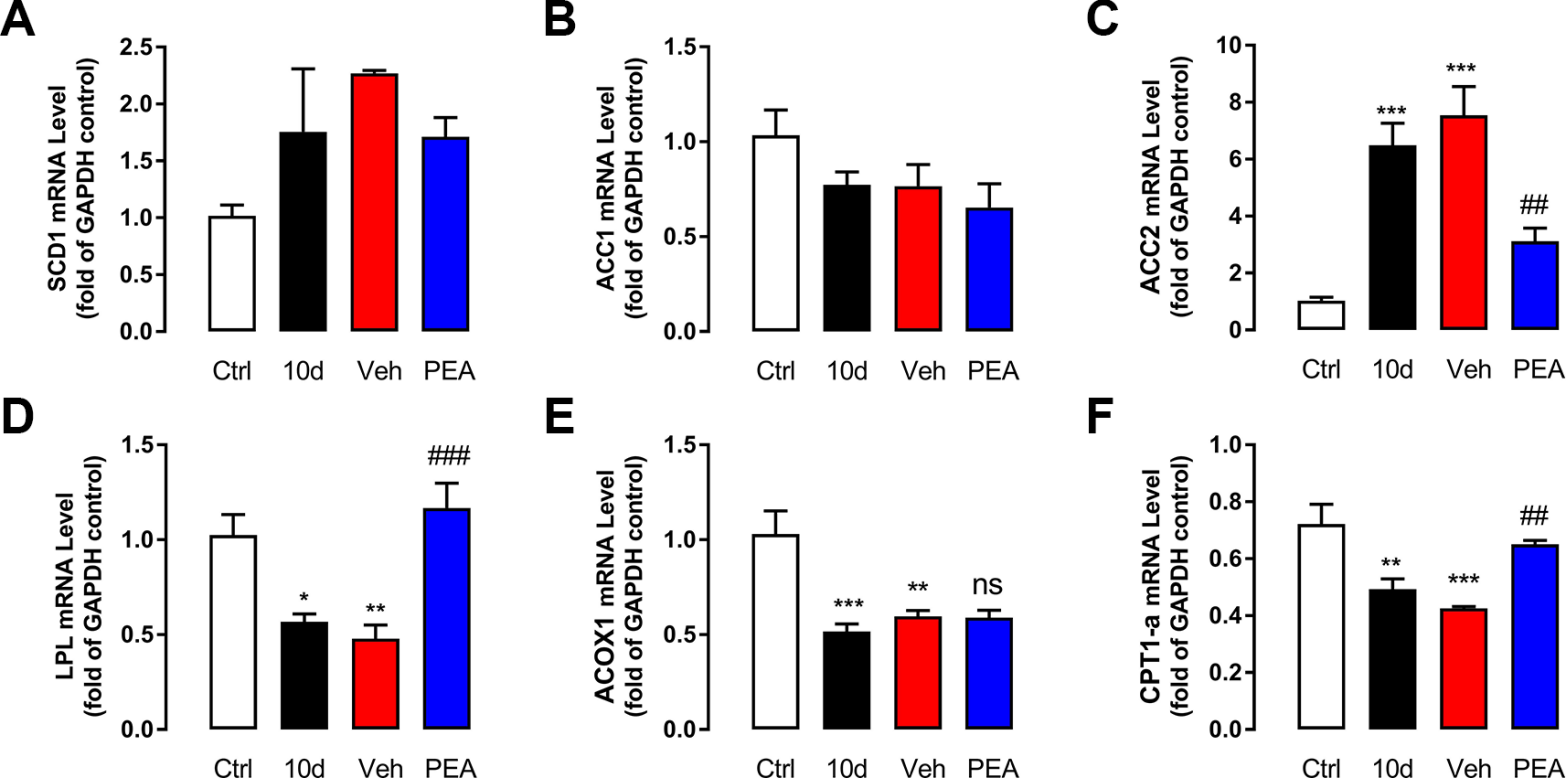
PEA administration normalized the mRNA levels related with lipid metabolism. The effects of control (blank), SD (model), vehicle (solvent), and PEA (30 mg/kg) on mRNA expression levels of SCD1 **(A)**, ACC1 **(B)**, ACC2 **(C)**, LPL **(D)**, ACOX1 **(E)**, CPT1-α **(F)** in mouse LG. ns, not significant; *, P < 0.05, **, P < 0.01, ***, P < 0.001 vs. control group; ^##^, P < 0.01; ^###^, P < 0.001 vs. vehicle group, one-way ANOVA followed by Dunette’s *post hoc*, n = 5.

To determine the detailed molecular mechanism underlying the effects of PEA treatment on lipid metabolism, expression of lipogenesis-related genes (*SCD1, ACC1*, and *ACC2*), fatty acid oxidation–related genes (*CPT-1α* and *ACOX1*), and the triglyceride hydrolysis–related gene LPL in LG tissue were measured using RT-PCR (**Figure 4**). In the SD-induced group, mRNA expression of *ACC2* gene expression was notably higher in the SD group compared with the control group (**Figure 4C**). *LPL* and *CPT-1α* were significantly lower compared with the control group (**Figures 4D, F**). PEA treatment rescued gene expression levels of *LPL* and *CPT-1α* and suppressed the mRNA expression of *ACC2*. Other genes involved in lipid metabolism (e.g., *SCD1, ACC1*, and *ACOX1*) were unaffected after PEA administration.

### PEA Normalized Morphological Feature Changes in the LG and Acinar Cells

**Figure 5:**
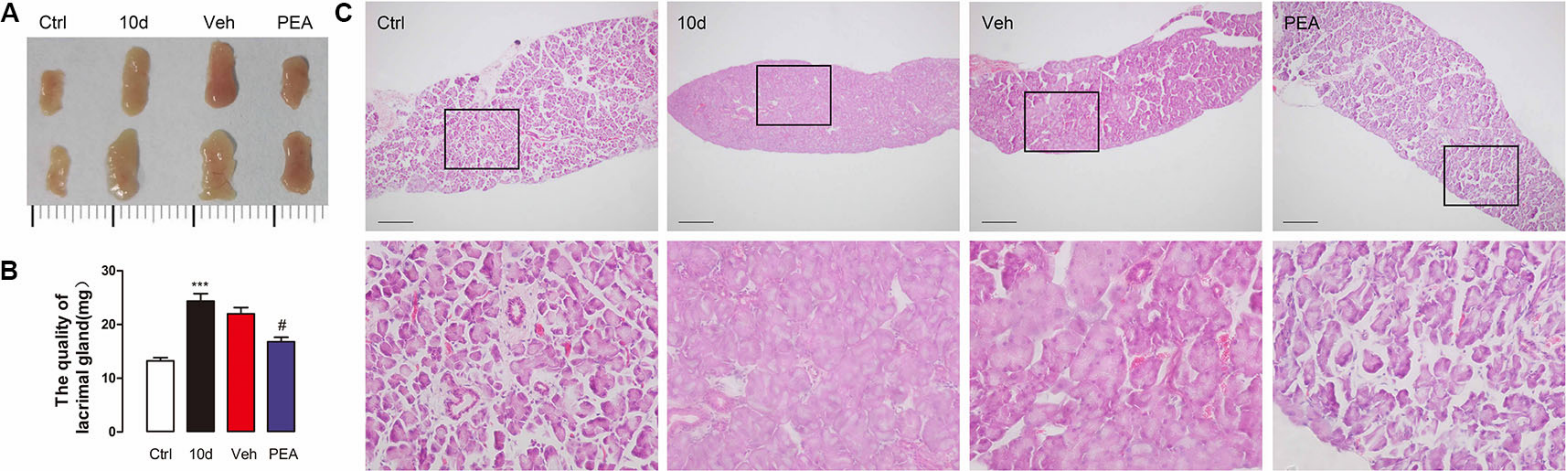
PEA normalized the morphological feature of LG. The increased size **(A)**, weight **(B)**, and acini size **(C)** of LG returned to normal levels after PEA treatment. ***, P < 0.001 vs. control group; ^#^, P < 0.05 vs. vehicle group, one-way ANOVA followed by Dunette’s *post hoc*, n = 5.

As mentioned above, the LG tissues were hypertrophic after the mice were exposed to SD for 10 days. The size (0.4 × 0.8 cm for 10-day SD mice vs. 0.3 × 0.5 cm for control group mice, **Figure 5A**) and weight (24.4 ± 2.8 mg for 10-day SD mice vs. 13.2 ± 1.3 mg for control group mice, **Figure 5B**) of the LG cells had a two-fold increase, compared with the control group mice. PEA treatment restored the morphology (0.3 × 0.6 cm for PEA treatment group vs. 0.4 × 0.8 cm for vehicle group, **Figure 5A**) and weight (16.8 ± 1.6 mg for PEA treatment group vs. 22.0 ± 2.6 mg for vehicle group, **Figure 5B**) of LGs to near normal levels. The hematoxylin and eosin staining results also indicated that 30 mg/kg PEA administration prevented LG hypertrophy in SD-induced mice (**Figure 5C**). This result was consistent with the results of the gradient dose experiment.

**Figure 6:**
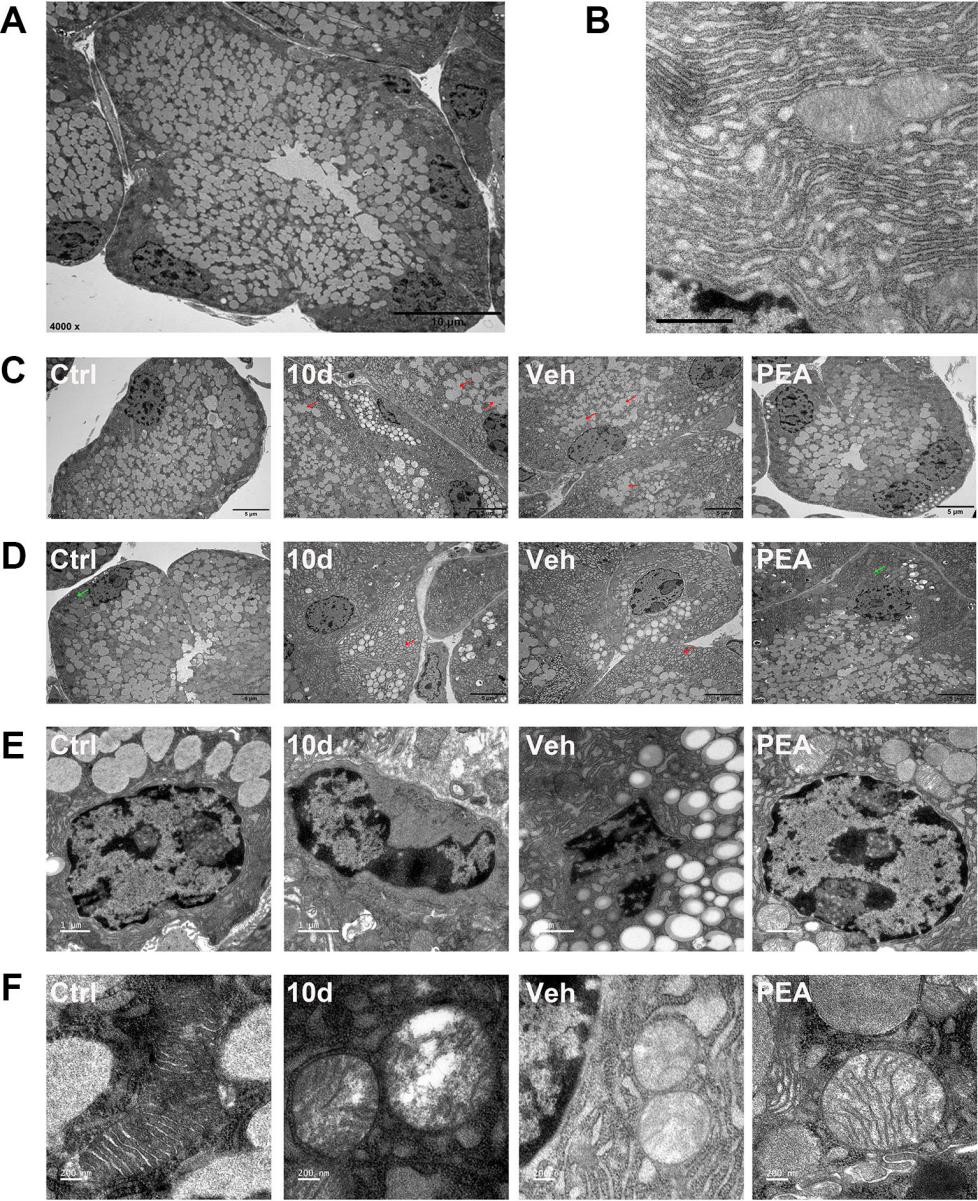
PEA normalized the ultrastructural features of LG. Representative transmission electron micrographs of normal acinar cell (**A** scale bars: 10 μm) and endoplasmic reticulum (ER, **B**, scale bars: 1 μm). SD induced secretory granules fusion (**C**, red arrows, scale bars: 5 μm), dilated cisternae of the ER (**D**, red arrows, scale bars: 5 μm), nuclear pyknosis (**E**, scale bars: 1 μm), and swollen mitochondria (**F**, scale bars: 200 nm) in LG acinar cell. PEA treatment restored the normal morphology secretory granules, ER (**D**, green arrows), nuclear and mitochondria. n = 3.

The LG ultrastructural changes represented an important feature of disorders of secretory function during progressively worsening effects of sleep loss (Vais et al., 2014). To examine PEA effectiveness, we used TEM to reveal the ultrastructural morphological features of the acinar cells. Most of the normal acinar cells were arranged in groups of five to seven cells and had the appearance of a rosette with a clear center (**Figure 6A**). Secretory granules were located close to the center of the cross-section of the rosette. SD decreased the numbers of secretory granules and induced fusion of isolated secretory granules in the acinar cells (**Figure 6C**). This result indicated that the tissue secretory activity was dysfunctional. The TEM revealed dilated endoplasmic reticulum with destroyed cisternae in the SD and vehicle groups (**Figure 6D**). In the PEA treatment group, the numbers of secretory granules and the morphology of the endoplasmic reticulum were similar to those of the control group mice; this result was consistent with the improvement in tear secretory function (**Figures 6B**–**D**). 

In the SD and solvent vehicle groups, a few acinar cells had irregular quadrilateral nuclei and nuclear pyknosis without disintegration (**Figure 6E**). Electron microscopy also revealed partially swollen mitochondria, apparent vacuolization, and loss of cristae in the acinar cells (**Figure 6F**). No nuclear pyknosis and destroyed mitochondria were found in the PEA treatment group; the cell nuclei were round and elliptical (**Figures 6E, F**). These results suggested that apoptosis might occur in the LG acinus after SD. PEA treatment normalized mitochondrial morphology and physiology and therefore rescued acinar cell fate.

### PEA Prevented Corneal Barrier Function Damage Caused by SD

**Figure 7:**
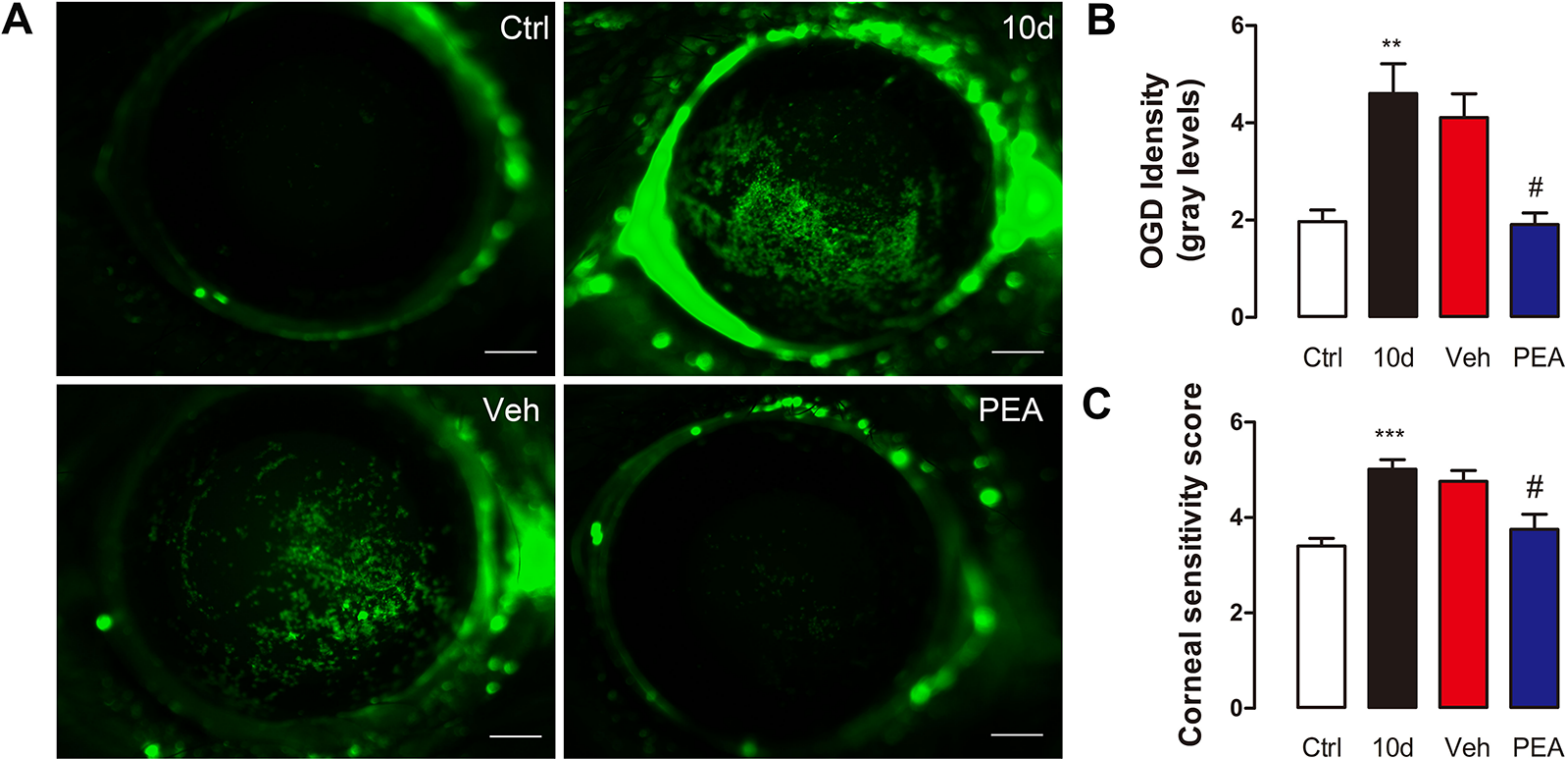
PEA reduced the corneal epithelial permeability and nerve sensitivity. Representative digital pictures of Oregon Green Dextran (OGD) corneas staining used to score corneal epithelial permeability **(A)**, scale bars: 500 μm. OGD intensity scores **(B)** and corneal sensitivity scores **(C)** were expressed as mean ± SD in non-treatment control group (Ctrl), sleep deprivation (SD) for 10d group (10d), SD for 10d + solvent vehicle group (Veh), and SD for 10d + 30 mg/kg PEA treatment group (PEA). **, P < 0.01, ***, P < 0.001 vs. control group; ^#^, P < 0.05 vs. vehicle group, one-way ANOVA followed by Dunette’s *post hoc*, n = 5.

Corneal epithelial damage is a significant DED-associated change. SD mice have signs of ocular surface damage that is similar, to some extent, to DED (Li et al., 2018). Corneal epithelial permeability was assessed *via* an ocular surface fluorescence staining test using the 70 kD high-molecular-weight fluorescent molecule, OGD. The OGD analysis of the ocular surface in the control mice revealed an intact, unbroken corneal barrier. In the SD and solvent vehicle group mice, the ocular surface had tissue injury that was revealed by the OGD uptake and corneal fluorescein staining scores (**Figure 7A**). Systemic application of PEA (30 mg/kg, i.p. route) markedly decreased ocular surface OGD intensity. This result suggested that the corneal epithelial damage was repaired (**Figures 7A**, **B**).

Abundant small sensory nerve endings that are located in the cornea epithelium respond to contact, temperature, and drying changes. We measured corneal nerve sensitivity in mice of the same age using a Cochet-Bonnet esthesiometer. As expected, the mean corneal sensitivity after 10 days of SD (5.0 ± 0.6 mm) was significantly higher than that of control group (3.4 ± 0.4 mm). This result was consistent with the result of a previous study (Bottemanne et al., 2018). PEA treatment normalized the corneal sensitivity to mechanical stimulation (3.7 ± 0.9 mm); the vehicle solvent (4.8 ± 0.6 mm) did not result in similar changes (**Figure 7C**). These results indicated that PEA treatment contributed to the maintenance of corneal homeostasis.

### PEA Increased the Number of Conjunctival Goblet Cells and Tear Secretion

**Figure 8:**
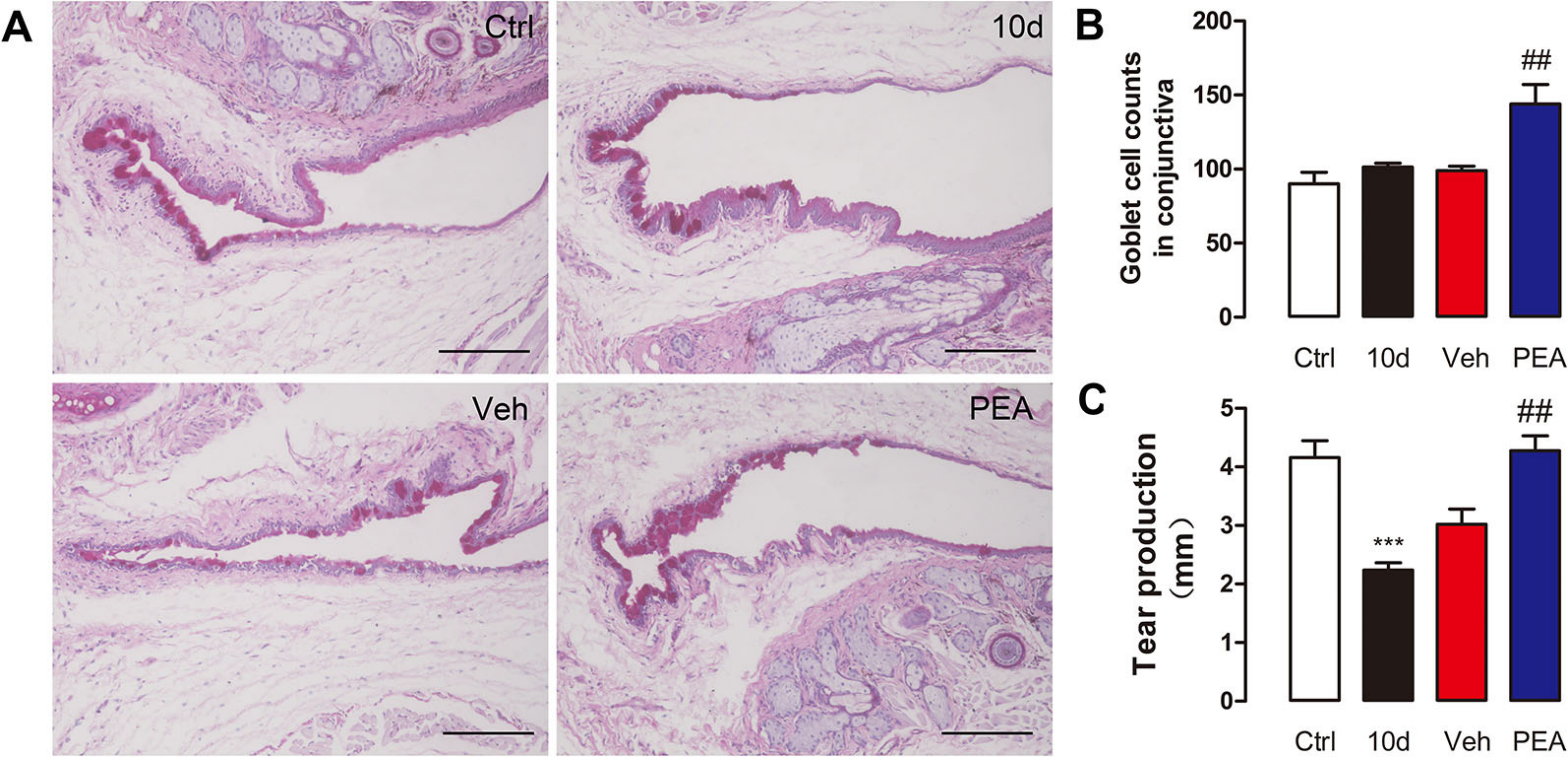
PEA increased conjunctival goblet cells number and tear secretion. Representative images of PAS conjunctivae staining **(A)**, scale bars: 150 μm. Goblet cell density **(B)** and tear production **(C)** were expressed as mean ± SD in non-treatment control group (Ctrl), sleep deprivation (SD) for 10d group (10d), SD for 10d + solvent vehicle group (Veh), and SD for 10d + 30 mg/kg PEA treatment group (PEA). ***, P < 0.001 vs. control group; ^##^, P < 0.01 vs. vehicle group, one-way ANOVA followed by Dunette’s *post hoc*, n = 5.

Conjunctival goblet cells are important for tear secretion and tear film stability *via* secretion of gel-forming mucins. The periodic acid–Schiff staining results indicated that SD slightly increased the mean number of goblet cells, compared with control group (101 ± 7 vs. 90 ± 25), but the difference was not statistically significant (**Figures 8A**, **B**). This change in goblet cells is an adaptive response to the decreased tear secretion at the early stage of DED (Li et al., 2018). Application of PEA markedly increased conjunctival goblet cell density (144 ± 29) (**Figure 8C**). However, the detailed mechanisms associated with goblet cell differentiation during PEA treatment require further investigation.

The phenol red thread test results indicated that phenol thread wetting was notably decreased after 10 days of SD (from 4.2 ± 1.0 to 2.2 ± 0.4 mm). After 5 days of PEA treatment, a significantly greater increase in phenol thread wetting was observed, compared with the solvent treatment group (4.3 ± 0.8 mm for PEA vs. 3.0 ± 0.8 mm for vehicle) (**Figure 8C**). These results indicated that PEA improved lacrimal secretory system function.

### PPAR-α Mediates the Effect of PEA on SD-Induced Lacrimal Damage and Dry Eye Syndrome

PEA is an endogenous agonist of PPAR-α. In a previous study, we found that SD-induced declines in corneal PPAR-α expression result in increased corneal lipid accumulation (Tang et al., 2018). We also found that PPAR-α^−^^/^^−^ mice have very similar corneal lipid accumulation compared to SDE mice. In this study, we compared the LGs of PPAR-α^−^^/^^−^ mice and SDE mice. Loss of PPAR-α function also resulted in similar signs, including LG cell hypertrophy, acinar cell enlargement, lipid accumulation, and ocular epithelial barrier function damage. This result was consistent with the changes that occurred in the SD group.

**Figure 9:**
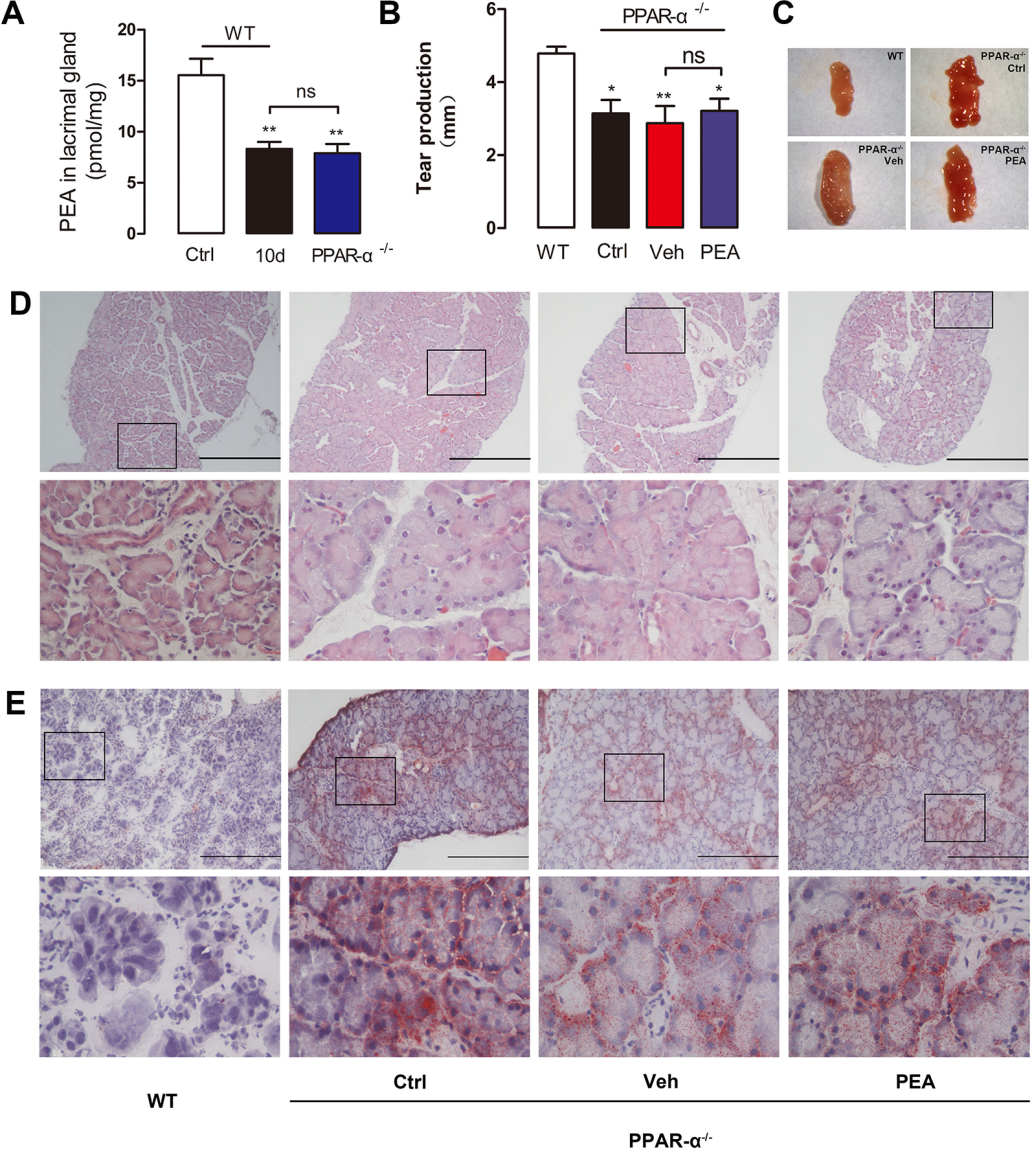
Effects of PEA on PPAR-α*^−^* ^/^ *^−^* mice. The PEA levels in LGs of PPAR-α*^−^* ^/^ *^−^* mice decreased markedly comparing with WT mice **(A)**. PEA could not increase the tear production **(B)**, reduce the LG **(C)** and acinar cell size **(D)**, and ameliorate lipid deposition **(E)** in PPAR-α*^−^* ^/^ *^−^* mice. *, P < 0.05, **, P < 0.01 vs. control group, one-way ANOVA followed by Dunette’s *post hoc*, n = 5.

The difference in LG endogenous PEA concentration between the PPAR-α^−^^/^^−^ mice (8.3 ± 1.7 pmol/mg) and the SDE mice (8.32 ± 1.68 pmol/mg, 10 days of SD) was not statistically significant. Their PEA levels were markedly decreased, compared with the wild-type group (15.55 ± 2.77 pmol/mg, **Figure 9A**). Exogenous PEA was administered to PPAR-α^−^^/^^−^ mice to examine the detailed molecular mechanisms involved with PEA protection of SDE. Tear secretion of PPAR-α^−^^/^^−^ mice (3.1 ± 1.0 mm) was significantly lower than that of wild-type mice (4.8 ± 0.5 mm). However, treatment with 30 mg/kg PEA (3.2 ± 0.9 mm) did not result in increased tear secretion in the PPAR-α^−^^/^^−^ mice. This group had a response that was similar to that of the vehicle solvent group (2.9 ± 1.3 mm, **Figure 9B**). Similarly, application of exogenous PEA did not reduce the size of the enlarged LGs in the PPAR-α^−^^/^^−^ mice (**Figure 9C**). The hematoxylin and eosin staining results suggested that after PEA treatment, acinar cell size and vesicle number remained similar to the vehicle solvent group (**Figure 9D**). The ORO staining results indicated that severe neutral lipid accumulation was located close to the cell nucleus in the PPAR-α^−^^/^^−^ mice; PEA treatment did not reduce the lipid deposition (**Figure 9E**). These results suggested that the PEA-associated improvements in lipid deposition, LG cell hypertrophy, and dry eye signs in the SDE mice were partly associated with the PPAR-α receptor.

Since PPAR-α^−^^/^^−^ mice cannot tolerate the “stick over water” test for extended periods, the SD process cannot be modeled using exposure of these mice to this test. For the PPAR-α^−^^/^^−^ mouse models, the dry eye symptoms were induced via loss of PPAR-α function instead of via SD. To further validate that PEA efficacy in the SDE mice depended on the involvement of the PPAR-α nuclear receptor, the specific PPAR-α antagonist, MK886, was used for further investigation. When mice were pre-treated with MK886, the PEA-associated improvement in LG was completely abolished.

**Figure 10:**
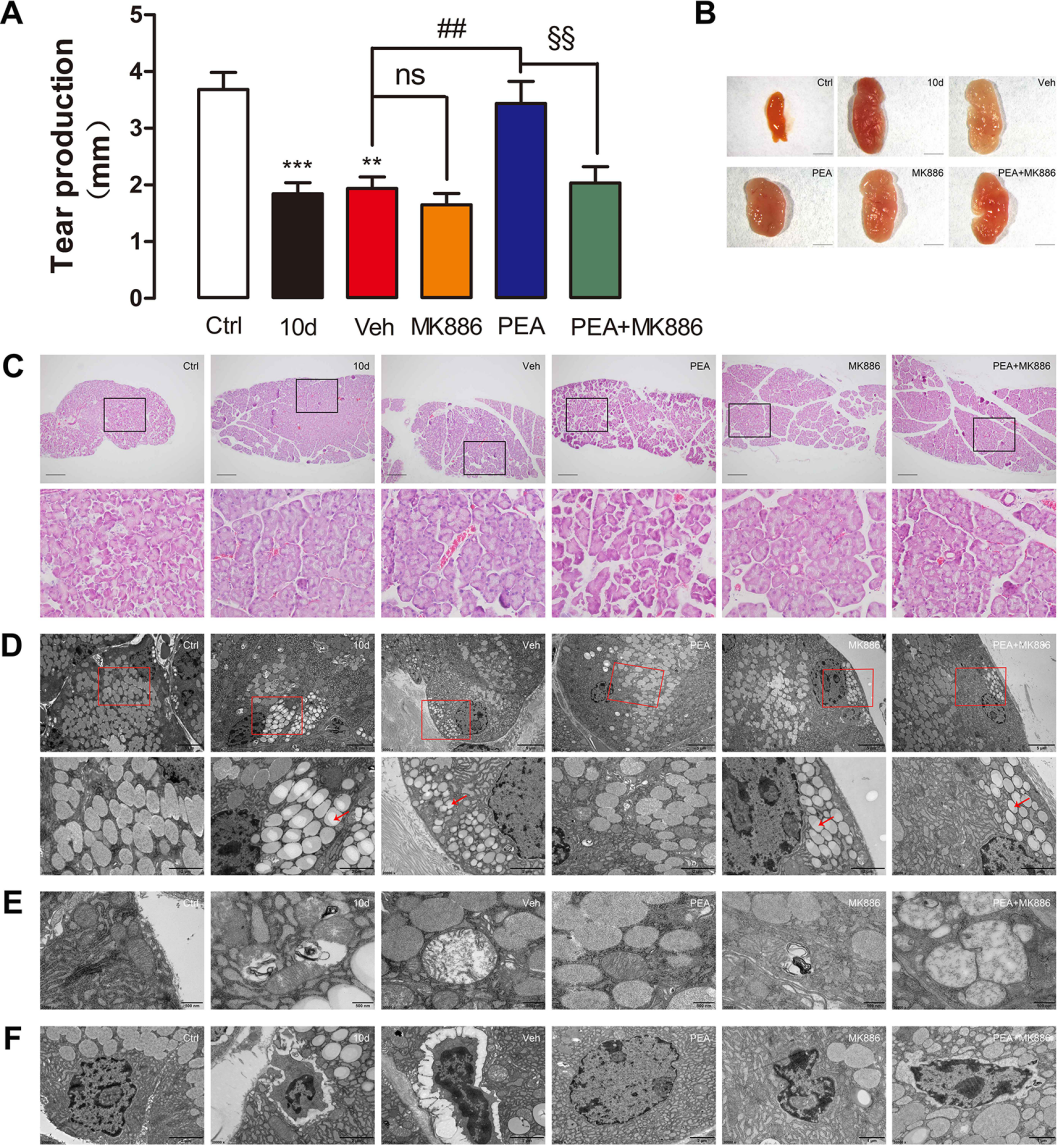
Pretreatment with PPAR-α antagonist MK886 blocked the effects of PEA on SD-induced mice LG. MK886 blocked tear production **(A)**, normalization of LG **(B)** and acinar cell size **(C)** rescued by PEA in SD-induced mice. Transmission electron microscope (TEM) observation indicated that the effects of PEA against lipid accumulation, mitochondrial swelling, and nuclear pyknosis were completely abolished by pretreatment of MK886 **(D–F)**. **, P < 0.01, ***, P < 0.001 vs. control group; ns, not significant, ^##^, P < 0.01 vs. vehicle group; ^§§^, P < 0.01 vs. PEA group; one-way ANOVA followed by Dunette’s *post hoc*, n = 5.

Tear secretion in the SD group (1.8 ± 0.8 mm) was significantly lower than that of the control group mice (3.7 ± 1.1 mm), but PEA application resulted in a return to normal levels of tear secretion (3.4 ± 1.6 mm, **Figure 10A**). Treatment with MK886 only (1.7 ± 0.7 mm) and coadministration of MK886 and PEA (2.0 ± 1.1 mm) did not elevate the tear secretion levels markedly, compared with the SDE mice (**Figure 10A**). Similarly, MK886 prevented improvement after PEA was administered to mice with LG hypertrophy induced by SD (**Figure 10B**). The hematoxylin and eosin staining results indicated that the reduction in acini size caused by PEA was blocked by MK886 treatment (**Figure 10C**). The electron microscopy results also revealed that lipid droplet accumulation (**Figure 10D**), mitochondrial swelling (**Figure 10E**), and nuclear pyknosis (**Figure 10F**) were present when MK886 was administrated with PEA.

## Discussion

Medications used clinically to treat DED mainly include artificial tears, anti-inflammatory drugs, immunosuppressant drugs, and mucin secretion promoters (Perez et al., 2016). Development of new drugs to treat DED is progressing very slowly because some problems remain unresolved (e.g., lack of a precise definition and classification, the specific pathogenesis, and diagnosis). Environmental stress, autoimmune disease, and aging are the most important risk factors for dry eye symptoms (Simsek et al., 2018). Other risk factors, which are associated with the increasing prevalence of DED, include video display terminal use, sleep deficits, and contact lens wear (Tan et al., 2015). However, treatment is based on a lack of complete information because the specific pathophysiological mechanisms of lifestyle-induced DED are incompletely understood.

Anxiety, staying up late, and abuse of video display terminals are negatively affecting amounts and quality of sleep. Inadequate sleep has gradually become a common problem of modern society and an important cause of dry eye symptoms that cannot be ignored. In this study, we used an SD mouse model to mimic the DED induced by sleep deficiency. A previous study found that the pathological changes affecting SDE mice include decreased aqueous tear secretion, defects and squamous metaplasia of corneal epithelial cells, and corneal barrier function damage (Li et al., 2018). We did not find obvious corneal inflammation after 10 days of SD. This result was very different from the results using other SDE mouse models induced by environmental stress, oxidative stress, and scopolamine (Li et al., 2018). The typical features of SDE mice are LG cell hypertrophy, abnormal superficial corneal epithelial cell microvillus morphology, and severe LG lipid accumulation (Li et al., 2018; Tang et al., 2018). In clinical practice, use of artificial tears and anti-inflammatory eye drops is not a significant and effective treatment approach for these populations. Based on the premise of improving sleep quality, restoration of eye tissue homeostasis is likely a possible way to treat sleep deficiency–induced DED.

We found that SD disrupted lipid homeostasis in the LG; endogenous PEA levels decreased in a time-dependent manner. The mechanism for this regulation was mainly attributed to a decrease in NAPE-PLD, which is the enzyme responsible for the synthesis of FAEs. We found that mRNA levels of NAAA, the specific hydrolase of PEA, remained constant during 10 days after SD. NAAA is almost exclusively expressed in immune cells (Bonezzi et al., 2016; Bottemanne et al., 2018) and is catalytically active at the acidic pH present during inflammation. In the SDE mouse model, no obvious inflammatory cell infiltration was found in ocular surfaces or LGs, which might explain why NAAA enzyme was not highly expressed. The expression of FAAH, the major hydrolase of AEA and OEA, reduced simultaneously with the NAPE-PLD decrease in SD mice. It means that the synthesis and degradation of AEA and OEA declines simultaneously, and the levels of AEA and OEA were unaffected. PEA is involved in the sleep–wake rhythm and has the highest values in the pons and hippocampus during the lights-off period of an experimental rat model. This result is consistent with the PEA reduction during the SD period. The physiological significance of the alteration of PEA levels remains unclear.

FAE is a class of endogenous lipids with a diversity of biological effects. PEA and OEA are both involved in the regulation of lipid metabolism (Ueda et al., 2010). OEA is a satiety factor that suppresses feeding and stimulates lipolysis by activating PPAR-α receptors and in turn engaging vagal sensory afferent nerves (Hankir et al., 2017; Igarashi et al., 2017). However, there is not enough evidence to confirm the effects of PEA on appetite regulation and weight gain. In 2014, Raso et al. reported that PEA reduces food intake, fat mass, and body weight in ovariectomized rats through improvement in hypothalamic leptin signaling (Mattace Raso et al., 2014). In this study, SD downregulated the PEA/PPAR-α signaling pathway in the LGs of experimental mice. This result suggested that there was a decrease in lipid peroxidation and an increase in neutral lipid deposition. Excess lipid in LG tissues resulted in acinar cell hypertrophy during the SD period. This morphological modification is unhealthy because normal acinar cells produce sufficient quantities of aqueous tears and glycoproteins (Dartt, 2009). PEA treatment normalized cell size and reduced the number of vesicles in the acinus, suggesting a benefit by maintaining lipid metabolism homeostasis in acinar cells. The ORO staining and TEM examination results further clarified the lipid content-lessening effects of PEA.

The causal relationship between lipid accumulation in LGs and dry eye symptoms and clinical signs remains unclear. Severe lipid deposition and compensatory hypertrophy in LGs occurs in non-obese diabetic mice (Wu et al., 2009); this change is likely due to impaired acinar cell lipid efflux. The LGs of male non-obese diabetic mice (12 weeks of age) have no noticeable tissue necrosis and no detectable immune cell infiltration (Wu et al., 2009). In our previous study, the lack of immune cell infiltration in the LGs of 10-day SD mice (Li et al., 2018) is consistent with the results in non-obese diabetic mice. Severe acinar cell damage that included reductions in dense-core secretory granules, disruption of endoplasmic reticulum structure, mitochondrial swelling, and pyknotic nuclei was found during the TEM analysis. Taken together, these results and the previous study results (Wu et al., 2009; Li et al., 2018) suggest that these LG dysfunctions are critical origins of the development and progression of DED in SDE mice. Accelerated lipid deposition in acinar cells is associated with LGD. PEA administration prevented lipid droplet deposition, cellular morphological changes, and LGD, and subsequent improvements on dry eye signs and ocular surface health.

PPAR-α is well-known for its role in fatty acid *β*-oxidation and lipid metabolism (Ramakrishnan et al., 2016). However, as the endogenous PPAR-α ligand, PEA has been proposed to mainly be an analgesic, neuroprotective, and anti-inflammatory agent in clinical trials and animal experiments (Gugliandolo et al., 2017). There are few reports on the relationships between PEA and lipid metabolism. Here, we measured mRNA expression of enzymes involved in lipid biosynthesis and degradation to elucidate the preliminary mechanisms underlying the effects of PEA treatment. Lipoprotein lipase has an important role during the hydrolysis of triglycerides, which is the rate-limiting step during supply of available free fatty acids and monoacylglycerols to tissues. The lipid-lowering efficacy of synthetic PPAR-α agonists fibrates is related to increased LPL expression and activity (Brautbar et al., 2012). Carnitine palmitoyltransferase 1α is the rate-limiting enzyme that catalyzes fatty acid *β*-oxidation in mitochondria; it is the typical downstream target of nuclear receptor PPAR-α (Ahn et al., 2014). Consistent with these findings, PEA enhanced the mRNA expression of LPL and CPT-1α in a PPAR-α–dependent manner, which would stimulate lipolysis and reduce fatty acid uptake in the mouse LGs. Acetyl-CoA carboxylase is a key enzyme involved in *de novo* fatty acid biosynthesis. The predominant isoform, ACC1, is distributed in adipose tissue, the liver, and mammary glands; ACC2 is highly expressed in the skeletal muscle, liver, and heart (Fullerton et al., 2013). We found that compared with the SD group, the mRNA expression of ACC2 in LG tissue decreased after PEA treatment; expression of ACC1 was unchanged. Although the detailed roles of ACC2 on whole-body energy metabolism remain undetermined (Choi et al., 2007; Hoehn et al., 2010), local decreased expression of ACC2 is beneficial for fat oxidation and reduction. Other genes involved in lipid balance (e.g., SCD1 and ACOX1) were unaffected by PEA treatment.

Experiments using PPAR-α^−^^/^^−^ mice and the PPAR-α antagonist MK886 further identified that exogenous PEA treatment inhibited excessive lipid droplet accumulation in LG tissue and regained LG function to improve the signs of dry eye on the ocular surface in a PPAR-α–dependent manner.

LGD is an underlying cause of dry eye syndrome, especially for people with long-term sleep deficiency. However, current treatment protocols are still insufficient. The results of this study suggested that targeting the PEA/PPAR-α pathway is a viable therapeutic option for the treatment of SDE syndrome. However, many questions remain to be answered. The LG most extensively receives parasympathetic innervation. However, only a few studies showing that SD decreases parasympathetic tone and thus may have an effect on the tear secretion and ocular surface (Lee et al., 2014). PPAR-α activation was identified to recruit local afferents of the vagus nerve, the major nerve of the parasympathetic division, in fat-induced mice (Campolongo et al., 2009). Although there is an abundance of literatures demonstrating that the endogenous PPAR-α agonist is a neurological modulator (Beggiato et al., 2019), there is no direct evidence that PEA regulates the parasympathetic nerve. SD elevates levels of stress hormones such as glucocorticoids and adrenaline and affects the immune system, which are also associated with the accumulation of lipids. Whether PEA regained LG lipid homeostasis by improving the above factors remains to be determined. Further experiments are needed to understand the underlying mechanisms of PEA treatment in SD-induced DED.

## Data Availability Statement

All data generated for this investigation are included in the article.

## Ethics Statement

All animal experimental protocols were performed in accordance with the guidelines of ARVO Statement for the Use of Animals in Ophthalmic and Vision Research, and approved by the Experimental Animal Ethics Committee of Xiamen University in China.

## Author Contributions

QC conducted most of the experiments and prepared the figures. CJ, RZ, YL, YH, and PZ conducted some of the experiments and performed animal studies. JR and LY provided suggestions and technical support for the project. ZL and YQ conceived and designed the experiments. ZL, YQ, and QC wrote and revised the manuscript.

## Funding

The present study was supported by the National Natural Science Foundation of China (no. 81603145), the National Key R&D Program of China (no. 2018YFA0107304), the Fujian Health-Education Research Grant (no. WKJ2016-2-03), and the Natural Science Foundation of Fujian Provincial (no. 2017J01146). We thank experimentalist Baoying Xie of Central Laboratory in the School of Medicine, Xiamen University for HPLC-MS2 technical support and data analysis.

## Conflict of Interest

The authors declare that the research was conducted in the absence of any commercial or financial relationships that could be construed as a potential conflict of interest.
